# Planar liquid crystal polarization optics for near-eye displays

**DOI:** 10.1038/s41377-021-00567-w

**Published:** 2021-06-08

**Authors:** Yan-qing Lu, Yan Li

**Affiliations:** 1 National Laboratory of Solid State Microstructures, Nanjing University, 210093 Nanjing, China; 2 Department of Electronic Engineering, Shanghai Jiao Tong University, 200240 Shanghai, China

**Keywords:** Displays, Liquid crystals

## Abstract

As a promising candidate for next-generation mobile platforms, virtual reality and augmented reality have the potential to revolutionize the way we perceive and interact with various types of digital information. In the meantime, ultrathin planar liquid crystal polarization optics are enabling a new evolutionary trend in near-eye displays. A recent invited review paper published in *eLight* provides an insightful review on liquid crystal optical elements and their applications toward AR and VR.

After several decades of extensive material research and development, device innovation, and heavy investment in advanced manufacturing technologies, active-matrix liquid crystal displays (LCDs), organic light-emitting diodes (OLEDs), and micro-LED displays have gradually displaced bulky cathode-ray tubes (CRTs) to become the dominant flat panel display technologies^1^. Their widespread applications cover smartphones, pads, notebooks, and desktop computers, TVs, data projectors, etc. In addition to displays, liquid crystals (LCs) exhibit other attractive properties, such as phase-only modulation and photopatternable characteristics. These properties can dynamically manipulate the wavefront of a light source and create new photonic devices, such as gratings and lenses. Recently, LC-based diffractive optical elements have attracted increasing interest due to their advantages such as high efficiency, polarization selectivity, ability to perform dynamic switching, and ultrathin form factor (several micrometers). In particular, the latter enables the development of very compact and lightweight optical components with electronic modulation capacity compared to their counterparts using mechanical modulation. These unique properties can be employed to address the major challenges in augmented reality (AR) and virtual reality (VR) displays and enable the novel photonic application.

With the steady development of high-speed communication and computation, the desire for a deeper level of visual interaction with the digital world beyond flat panel displays has led to the emergence of near-eye displays, such as those utilized by VR and AR (Fig. 1a). Both VR and AR aim to deliver high-quality 3D images to the viewer’s eyes. The major difference is that VR is immersive, while AR enables users to see through the real world. Unlike flat panel displays, the research of which is focused on the display panel itself, near-eye displays involve more complicated optical system engineering to satisfy the demanding requirements of the human eye, such as visual acuity, field of view, eye box, and 3D cues. In the meantime, the display system should have a favorable glasses-like form factor and be lightweight for a better wearing experience.

**Fig. 1:**
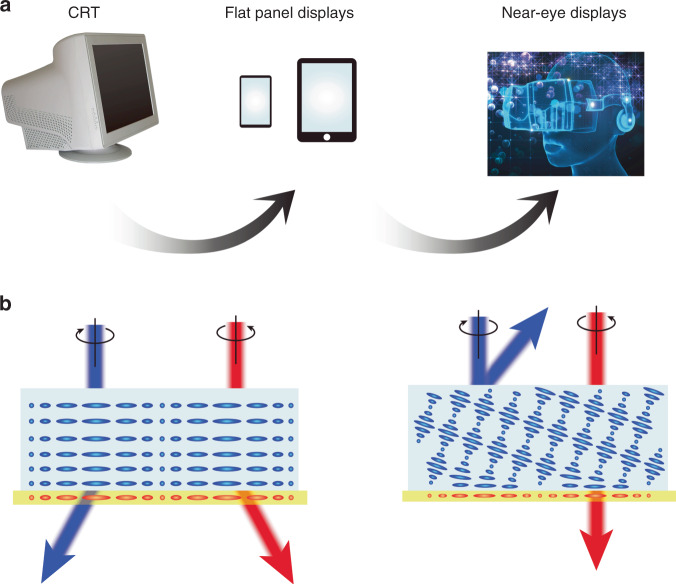
**a** Evolution of display technologies. **b** Polarization-dependent response of LCOEs (some images in **a** are purchased from a stock image service and have no copyright issues)

It is straightforward to realize that the abovementioned challenges are difficult to overcome in the short-term. More often than not, trade-off relations exist among several factors in the system design. Even with well-developed designs and the fabrication of geometric optics, we are often constrained by fundamental physical limitations, such as the conservation of optical invariants^2^. New light modulation methods beyond traditional optics are in urgent need.

Research on liquid crystal optical elements (LCOEs) with photoalignment techniques began in the early 1980s^3^. Since then, recording materials and methods have been steadily improved. Most of these LC holographic optical elements are transmission-type elements. Until recently, reflective LCOEs have been developed^4^. Such reflective LCOEs use cholesteric liquid crystals combined with photoalignment techniques, but they exhibit relatively small diffraction angles. Later, large-angle reflective LCOEs were demonstrated, and investigation of the LC molecular distribution^5^ revealed the underlying reason for the LC stability to endure large diffraction angles. A rigorous simulation method^6^ has also been developed for analyzing the optical properties of LCOEs. The inherent LC optical anisotropy results in LCOE’s strong polarization dependency on the input light, which, combined with the LC active polarization modulation ability, provides dynamic control of the output optical functions (Fig. 1b).

Transmissive LCOEs, integrated with a fast-response LC material, can achieve advanced modulation abilities, such as those of beam steering and tunable-focus lenses. The latter can be applied in VR displays to mitigate a major challenge called the vergence-accommodation conflict for 3D image generation. The advent of reflective LCOEs coincides with the emergence of AR, which often requires reflective optical elements with good transparency. The versatile functions and active modulation of LCOEs enable them to act as key components in VR and AR systems to tackle the critical challengers.

A recent invited review paper in *eLight*^7^, entitled “Planar liquid crystal polarization optics for augmented reality and virtual reality: from fundamentals to applications”, by Jianghao Xiong and Shin-Tson Wu from the University of Central Florida, provides a timely and comprehensive review of LCOEs and their applications towards AR and VR. Starting from fundamental LC dynamics, the review explains the formation mechanism of different LCOEs, including transmissive and reflective types. Then, comprehensive optical simulation methods, including the 4 × 4 Jones matrix method and the rigorous coupled-wave analysis, are introduced. The optical properties of different types of LCOEs are elaborately described. Finally, the article enumerates several examples of how LCOEs are used to solve the major challenges of VR and AR systems, such as vergence-accommodation conflict, field of view, and form factor. With the advantages including high efficiency, compact form factor, dynamic switching ability, polarization-dependent behavior, and high degrees of design freedom, the ultrathin planar liquid crystal polarization optics would enable a new round of evolution in near-eye displays.
